# Effects of simvastatin on cell viability and proinflammatory pathways in lung adenocarcinoma cells exposed to hydrogen peroxide

**DOI:** 10.1186/2050-6511-15-67

**Published:** 2014-11-29

**Authors:** Luca Gallelli, Daniela Falcone, Monica Scaramuzzino, Girolamo Pelaia, Bruno D’Agostino, Maria Mesuraca, Rosa Terracciano, Giuseppe Spaziano, Rosario Maselli, Michele Navarra, Rocco Savino

**Affiliations:** Department of Health Science, University of Catanzaro, Catanzaro, Italy; Department of Medical and Surgical Sciences, University of Catanzaro, Catanzaro, Italy; Department of Experimental Medicine, University of Catanzaro, Catanzaro, Italy; Department of Experimental Medicine-Section of Pharmacology, School of Medicine, Second University of Naples, via Costantinopoli 16, 80136 Naples, Italy; Department of Drug Sciences and Health Products, University of Messina, IRCCS centro neurolesi “Bonino-Pulejo”, Messina, Italy

**Keywords:** Lung cancer, NF-κB, Matrix metalloproteinases, Innate immunity, IL-8, Simvastatin

## Abstract

Lung cancer is characterized by a high mortality rate probably attributable to early metastasis. Oxidative stress is involved in development and progression of lung cancer, through cellular and molecular mechanisms which at least in part overlap with proinflammatory pathways. Simvastatin is a statin with pleiotropic effects that can also act as an anti-oxidant agent, and these pharmacologic properties may contribute to its potential anti-cancer activity. Therefore, the aim of this study was to evaluate, in the human lung adenocarcinoma cell line GLC-82, the effects of a 24-hour treatment with simvastatin on hydrogen peroxide (H_2_O_2_)-induced changes in cell viability, ERK phosphorylation, matrix metalloproteinase (MMP) expression, innate immunity signaling, NF-κB activation and IL-8 secretion. Cell counting was performed after trypan blue staining, cell proliferation was assessed using MTT assay, and apoptosis was evaluated through caspase-3 activation and Tunel assay. Western blotting was used to analyze protein extracts, and IL-8 release into cell culture supernatants was assessed by ELISA. Our results show that simvastatin (30 μM) significantly (P <0.01) inhibited the proliferative effect of H_2_O_2_ (0.5 mM) and its stimulatory actions on ERK1/2 phosphorylation, NF-κB activation and IL-8 production. Furthermore, simvastatin decreased H_2_O_2_-mediated induction of the cellular expression of MMP-2 and MMP-9, as well as of several components of the signaling complex activated by innate immune responses, including MyD88, TRAF2, TRAF6 and TRADD. In conclusion, these findings suggest that simvastatin could play a role in prevention and treatment of lung cancer via modulation of important proinflammatory and tumorigenic events promoted by oxidative stress.

## Background

The respiratory system is remarkably susceptible to oxidative stress because of its peculiar anatomical and functional properties, mainly related to the large area exposed to the external environment. Therefore, the cellular/tissue injury triggered by the oxidant burden generated by air pollutants in association with cigarette smoking plays a pivotal role in the pathogenesis of several inflammatory and proliferative lung disorders, including chronic obstructive pulmonary disease (COPD), asthma, acute respiratory distress syndrome (ARDS), idiopathic pulmonary fibrosis (IPF), cystic fibrosis, and also lung cancer.

In particular, inhaled oxidants such as ozone and nitrogen dioxide cause sequestration of inflammatory cells into the pulmonary microcirculation, thus leading to their accumulation within air spaces. Cigarette smoke, which contains many oxidants and free radicals in both its gaseous and particulate phases [1], significantly contributes to recruit macrophages into the airways, as well as to increase neutrophil numbers within lung microvessels. Once recruited and activated, macrophages, neutrophils and eosinophils produce and release reactive oxygen species (ROS) such as hydroxyl radicals and superoxide anion (O_2_.^-^), the latter being rapidly converted to hydrogen peroxide (H_2_O_2_) by superoxide dismutase (SOD) [2]. ROS may interfere with signal transduction pathways regulating the functions of transcription factors such as nuclear factor κB (NF-κB) and activator protein-1 (AP-1) [3]. NF-κB and AP-1 are responsible for the coordinated expression of several genes that control inflammation, cell proliferation and apoptosis. Within this context a key role is played by mitogen-activated protein kinases (MAPK), whose targets are mainly represented by nuclear transcription factors, also including those involved in oxidative stress. In particular, the ERK1/2 subgroup of MAPK is activated by a MAPK kinase kinase named Raf (most commonly Raf-1), whose activation in turn requires the GTP-bound form of Ras family proteins [4]. Once activated, Raf-1 phosphorylates the MAPK kinases MEK1 and MEK2, that finally stimulate ERK1 and ERK2.

Airborne pollutants and cigarette smoke can induce the bronchial epithelium to acquire a proinflammatory phenotype, characterized by an increased production of autacoids, cytokines, and chemokines [5]. Oxidant-induced phenotypic changes may thus significantly contribute to the key pathogenic role played by bronchial epithelial cells in inflammatory airway disorders such as asthma and COPD. Moreover, ROS may also contribute via several different signalling pathways, including MAPK activation, to development and progression of lung cancer [6].

Lung cancer is the leading cause of neoplastic death worldwide. More than 80% of lung cancer cases belong to the non small cell lung cancer (NSCLC) type, which can be further subdivided into adenocarcinoma (approximately 40% of all NSCLCs), squamous cell carcinoma and large cell carcinoma [7]. The metastatic potential of NSCLC strongly correlates with the cellular expression of matrix metalloproteinases (MMPs), which are regulated by NF-κB and by the metastasis suppressor RECK (reversion-inducing-cysteine-rich protein with kazal motif) [8, 9]. Moreover, the complex cellular and molecular mechanisms underlying the development and progression of NSCLC are also significantly affected by the innate immune system [6]. Indeed, the latter represents the first line of defense against noxious agents such as ROS, which can damage the airway epithelium.

The poor survival rate of patients with NSCLC is mostly due to its high metastatic potential and also to a relative drug resistance [7]. Therefore, new and more effective pharmacological treatments for NSCLC are strongly needed. In this regard, increasing attention is currently being paid to statins because of their capability of inhibiting 3-hydroxy-3-methylglutaryl coenzyme A (HMG-CoA) reductase, which is the rate limiting enzyme within the mevalonate pathway. Hence, by blocking the synthesis of mevalonate and its isoprenoid derivatives farnesyl pyrophosphate (FPP) and geranyl geranyl pyrophosphate (GGPP), statins also prevent prenylation-dependent activation of oncogenic Ras proteins [10]. Indeed, statins may have a cytostatic effect on cancer cells, and can prolong the survival of cancer patients [11]. They also act as anti-oxidant, anti-inflammatory and anti-angiogenic factors, and could therefore prevent or inhibit cancer cell growth [12]. Furthermore, we have recently shown that simvastatin is able to induce a decrease in cell proliferation and a significant increase of apoptosis in human NSCLC cell cultures [13], as well as to modulate Ras, MMP-2/9 and NF-κB activity in pulmonary neoplastic tissues obtained from patients undergoing therapeutic surgery for lung cancer [14].

On the basis of the above mentioned considerations, the aim of our present study has been to investigate, in the human lung adenocarcinoma cell line GLC-82 exposed to H_2_O_2_, the effects of simvastatin on cell viability and apoptosis, ERK phosphorylation, MMP2/9 and RECK protein expression, NF-κB nuclear content and interleukin-8 (IL-8) secretion. Moreover, we also assessed the effects of simvastatin on H_2_O_2_-induced changes in the expression of some important proteins of the signaling network underlying innate immune responses, including MyD88, tumor necrosis factor (TNF) receptor-associated factors (TRAF)2/6, and TNF receptor type 1-associated death domain protein (TRADD).

## Methods

### Reagents

The anti-caspase-3 monoclonal antibody E83-77 was purchased from Abcam (Cambridge, UK). Trypan blue was purchased from Sigma (St. Louis, MO, USA). Anti-phospho-ERK1/2, anti-NF-κB-p65, anti-RECK, anti-MyD88, anti-TRAF2, anti-TRADD, anti-TRAF6, anti-MMP2 and anti-MMP9 monoclonal antibodies were purchased from New England Biolabs (Beverly, MA, USA); an anti-(total)-ERK1/2 polyclonal antibody was commercially provided by Santa Cruz Biotechnology, Inc. (Santa Cruz, CA, USA). Purified crystalline powders of simvastatin were commercially obtained from Sigma (St. Louis, MO, USA), and then dissolved into refrigerated and light-protected DMSO stock solution.

### Culture and treatment of human lung cancer cells

GLC-82, a human lung adenocarcinoma cell line [13], was cultured at 37°C, 5% CO_2_, in Dulbecco’s modified Eagle’s medium (DMEM) supplemented with 10% FCS, penicillin 100 U/ml, streptomycin 100 μg/ml, and amphotericin B 25 μg/ml in a humidified 5% CO_2_ atmosphere. For each treatment, cells were plated in a 100-mm polystyrene dish (Falcon, Becton-Dickinson, Lincoln Park, NJ, USA) and ten ml of supplemented DMEM were then added. When GLC-82 cells grew to about 70% confluence, they were exposed for 2 hours to H_2_O_2_ (0.5 mM) and then cells were or not exposed for 24 hour to simvastatin (30 μM). The medium was not changed after treatment. The solvent employed to dissolve this drug was used as a control. After this period, the medium was removed for IL-8 assessment (see later), and cells were processed for protein extraction and immunoblotting.

### Cell viability and proliferation

Cell viability was assessed by light microscopy and dye exclusion, using Trypan blue. Cell numbers were evaluated by direct counting using a hemocytometer, in agreement with our previous reports [15–17]. Cell proliferation was investigated by 3-[4,5-dimethylthiazol-2-yl]-2,5 diphenyl tetrazolium bromide (MTT) assay, based on the conversion by mitochondrial dehydrogenases of the substrate containing a tetrazolium ring into blue formazan, detectable spectrophotometrically. The level of blue formazan was then used as an indirect index of cell density. Briefly, after treatment with either H_2_O_2_ and/or simvastatin, cells were exposed to MTT (5 μg/mL) for 150 min at 37°C. The medium was then removed and cells were solubilized with acidified isopropanol and 2% sodium dodecyl sulfate (SDS). After complete solubilization, presence of blue formazan was evaluated spectrophotometrically at a reference wavelength of 650 nm. All experiments were carried out in triplicate.

### Protein extraction and immunoblot analysis

Following treatment with simvastatin, cells were lysed for Western blotting in radioimmunoprecipitation assay (RIPA) buffer, as previously described [18]. Moreover, nuclear extracts were obtained using the NE-PER cell fractionation kit (Thermo Scientific, Rockford, IL, USA). Briefly, whole cell lysates or nuclear proteins were then separated on a 12.5% sodium dodecyl sulfate-polyacrylamide gel electrophoresis (SDS-PAGE) and transferred onto polyvinylidene difluoride (PVDF) membranes (Amersham Pharmacia, Little Chalfont, UK). Immunoblotting was performed using the above mentioned monoclonal antibodies. After being “stripped”, membranes were re-probed with a polyclonal antibody against total (phosphorylated and unphosphorylated) ERK1/2 proteins. Antibody binding was visualized by enhanced chemiluminescence (ECL-Plus; Amersham Pharmacia); intensities of experimental bands were analyzed by computer-assisted densitometry and expressed as arbitrary units, as previously described [19]. These experiments were performed in triplicate.

### TUNEL assay

TdT (terminal deoxynucleotidyl transferase)-mediated dUTP nick-end labeling (TUNEL) assay was performed in agreement with our previous reports [13]. Briefly, cells were plated on 24 9 24 mm cover slips (Carlo Erba Reagenti, Milan, Italy) and placed in six-well microtitre plates (Corning Incorporated, Corning, NY, USA) in DMEM supplemented with 10% FCS, penicillin 100 U/ml, streptomycin 100 mg/ml, and fungizone 25 mg/ml; 24 h after plating, when cells had reached 50–60% confluence, they were treated or not with H_2_O_2_ (0.5 mM) for 2 hours, and then were exposed or not to simvastatin (30 μM) for 24 hours; the incubation medium was not changed. Following treatment, TUNEL assay was performed using MEBSTAIN Apoptosis TUNEL Kit Direct (MBL, Woburn, MA, USA), strictly following instructions provided by the manufacturer. Photographs were acquired using a Leica GRDM confocal microscope (Leica, Wetzlar, Germany).

### IL-8 secretion

Culture supernatants were collected and assayed for IL-8 by ELISA using a commercially available kit (Peli-Kine kit; Eurogenetics, Hampton, UK; sensitivity limit, 1 pg/ml), according to manufacturer’s protocol. These experiments were performed in triplicate.

### Statistical analysis

All data are expressed as mean ± standard error (SEM). Statistical evaluation of the results was performed by analysis of variance (ANOVA). Differences identified by ANOVA were pinpointed by unpaired Student’s t test. The threshold of statistical significance was set at P <0.05.

## Results

### Cell viability

**Figure 1:**
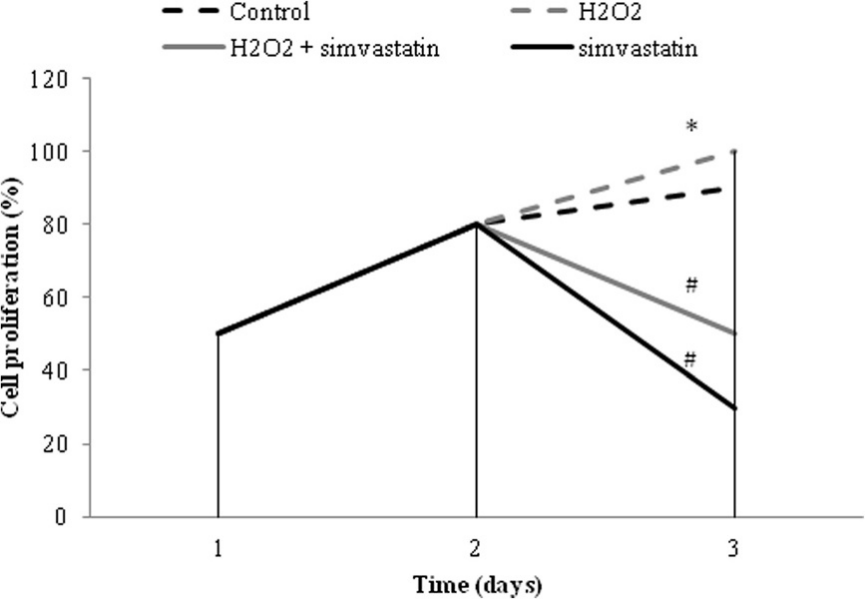
**Cell viability.** Effects of hydrogen peroxide (H_2_O_2_) on GLC-82 cell proliferation in the presence or absence of a 24 hours treatment with simvastatin (30 μM). Cell proliferation was assessed by MTT assay. ^*^P < 0.01 (H_2_O_2_ vs control); #P <0.01 (H_2_O_2_ + simvastatin and simvastatin vs H_2_O_2_). Data represent the mean of three experiments.

MTT assay detected an increase in cell number in cells pretreated with H_2_O_2_ (0.5 mM). In contrast, a 24 hours treatment with simvastatin (30 μM) was able to significantly decrease (P < 0.01) the cell number with respect to both control and pretreatment with H_2_O_2_. Therefore, simvastatin was able to modify normal growth cell, as well as to change the effect of H_2_O_2_ on cell proliferation (Figure 1).

### Caspase-3 activation

**Figure 2:**
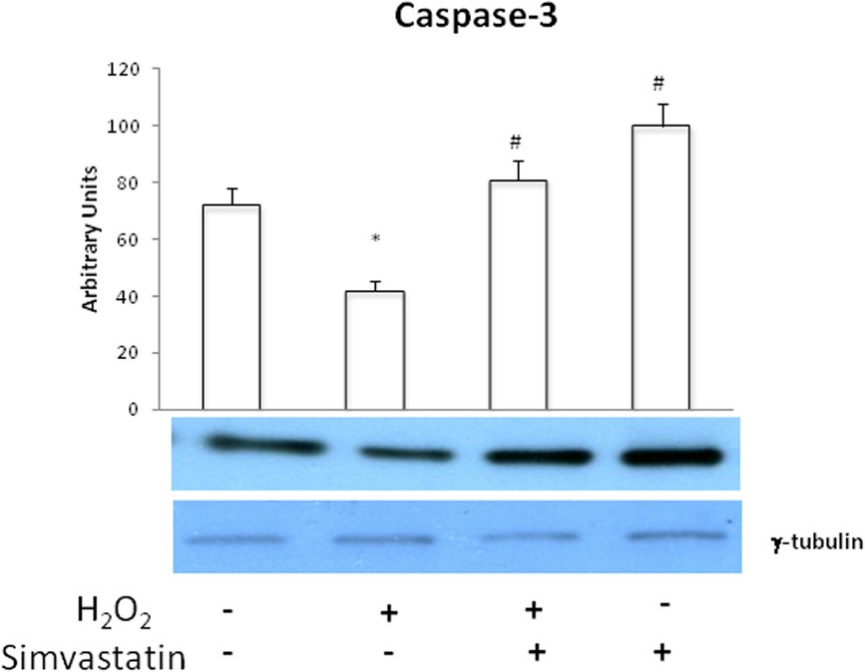
**Caspase-3 activation.** Western blot evaluation of caspase-3 expression with and without H_2_O_2_,in the presence or absence of a 24 hours treatment with simvastatin. Data represent the mean ± SEM of three experiments. **P <0.01. #P <0.01 (H_2_O_2_ + simvastatin and simvastatin vs H_2_O_2_).

Human GLC-82 cells were characterized by relatively low levels of caspase-3 activation, detected at baseline conditions as well as after exposure to H_2_O_2_ (0.5 mM) and after simvastatin treatment. Simvastatin (30 μM) significantly increased (P <0.01) the expression of active caspase-3 (Figure 2).

### TUNEL assay

**Figure 3:**
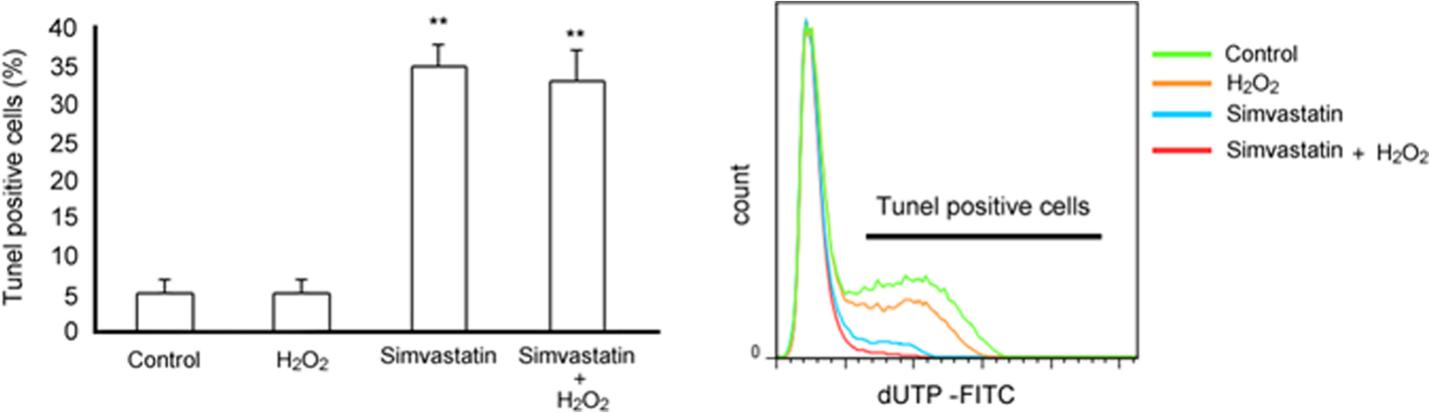
**TdT (terminal deoxynucleotidyl transferase)-mediated dUTP nick-end labeling (TUNEL) assay of GLC cells exposed or not to H**_**2**_ **O**_**2**_ **(0.5 mM) for 2 hours, and then to simvastatin (30 μM) for 24 hours.** ** *P* < 0.01 H_2_O_2_ + Simvastatin vs non treated cells (Cnt) and vs cells exposed to H_2_O_2_ (0.5 mM) alone.

H_2_O_2_ (0.5 mM) did not modify the number of TUNEL-positive cells, with respect to untreated cells (control). By contrast, in the presence of H_2_O_2_, simvastatin (30 μM) significantly (P < 0.01) increased (of about 30%) the number of TUNEL-positive cells (Figure 3).

### ERK phosphorylation

**Figure 4:**
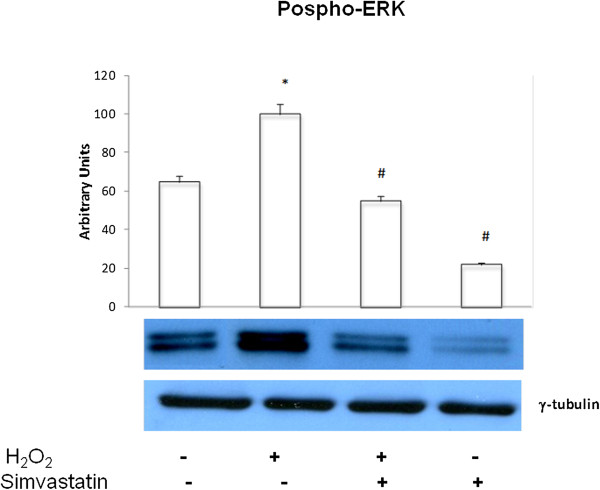
**ERK phosphorylation.** Western blot evaluation of phosphorylated ERK 1/2 (phospho-ERK) expression following or not H_2_O_2_ administration, and in the presence or absence of 24 hours treatment with simvastatin. Data represent the mean ± SEM of three experiments. *P < 0.01 (H_2_O_2_ vs control); #P < 0.01 (H_2_O_2_ + simvastatin and simvastatin vs H_2_O_2_).

Exposure of GLC-82 cells for 2 hours to H_2_O_2_ caused a significant (P < 0.01) increase in ERK1/2 phosphorylation; this effect was significantly inhibited in presence of a 24 hours treatment with simvastatin (P < 0.01) (Figure 4). Simvastatin (30 μM) induced a significant decrease (P < 0.01) of p-ERK expression. Both H_2_O_2_ and simvastatin exerted their effects uniquely on phosphorylation-dependent activation of ERK1/2, without affecting its total expression, as demonstrated by the unchanged binding patterns of the anti-(total)ERK polyclonal antibody (data not shown).

### Matrix metalloproteinase expression

**Figure 5:**
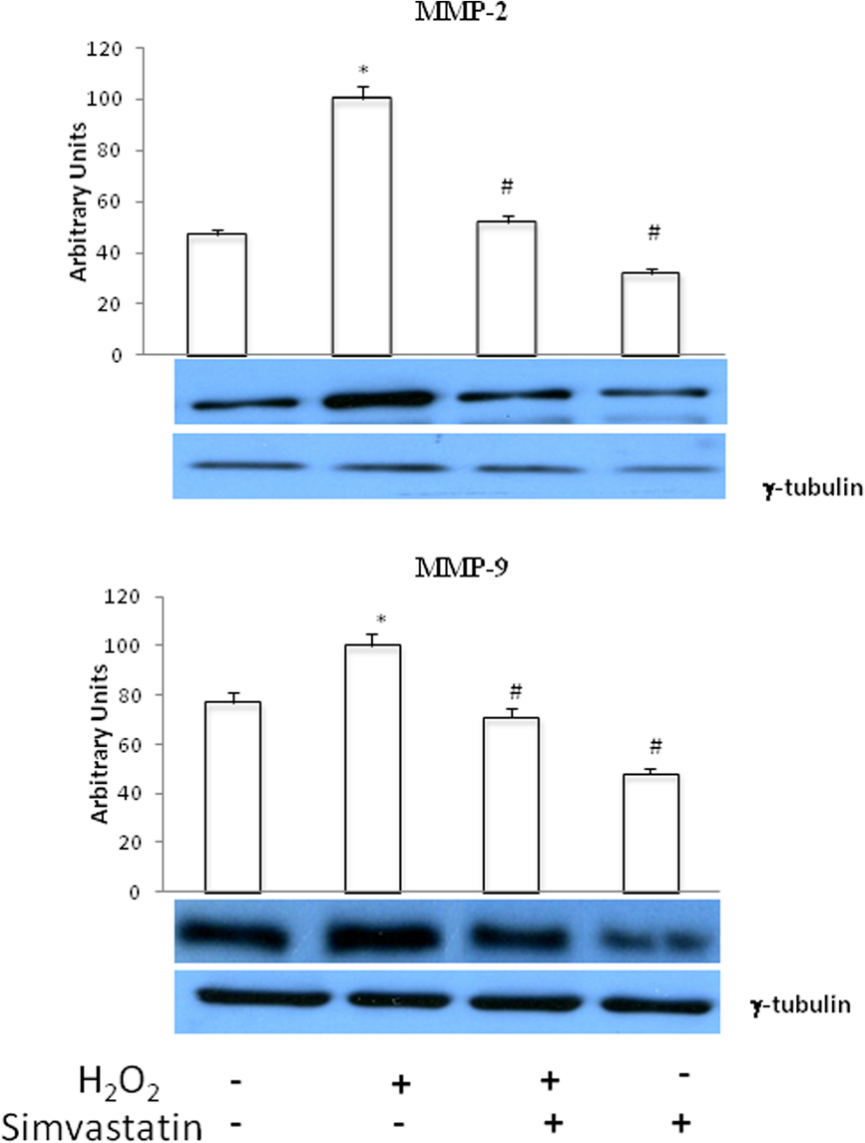
**Matrix metalloproteinase expression.** Western blot evaluation of MMP-2 (upper panel) and MMP-9 (lower panel) expression following or not H_2_O_2_ administration, and in the presence or absence of 24 hours treatment with simvastatin. Data represent the mean ± SEM of three experiments *P < 0.01 (H_2_O_2_ vs control); #P < 0.01 (H_2_O_2_ + simvastatin and simvastatin vs H_2_O_2_).

H_2_O_2_ induced a significant (P <0.01) increase in MMP-2 and MMP-9 expression. These effects were reverted by treatment with simvastatin (Figure 5). Moreover, simvastatin induced a significant decrease of both MMP-2 and MMP-9 baseline expression.

### RECK expression

**Figure 6:**
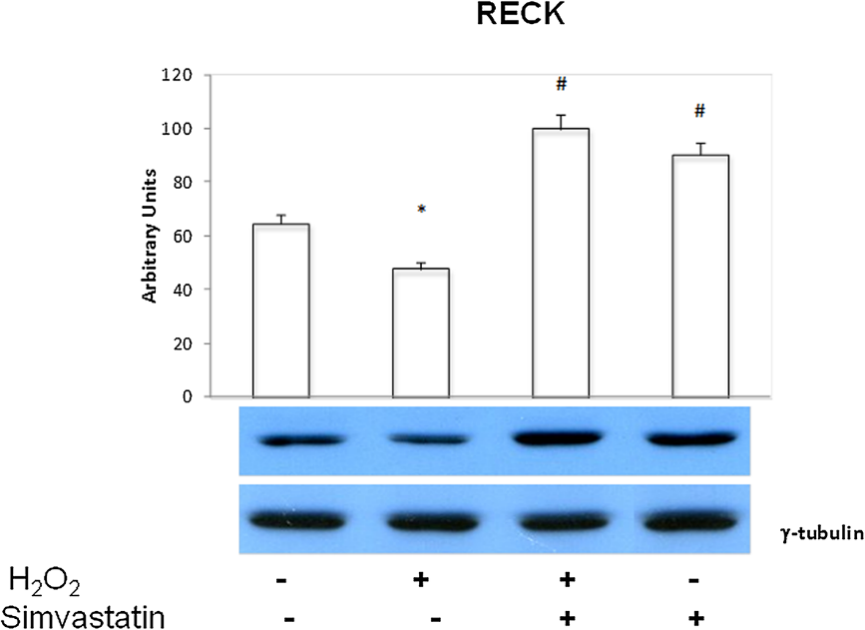
**RECK expression.** Western blot evaluation of RECK expression following or not H_2_O_2_ administration, and in the presence or absence of 24 hours treatment with simvastatin. Data represent the mean ± SEM of three experiments. *P < 0.01 (H_2_O_2_ vs control); #P < 0.01 (H_2_O_2_ + simvastatin vs H_2_O_2_).

H_2_O_2_ induced a significant (P <0.01) decrease in RECK expression, whereas treatment with simvastatin significantly (P <0.01) enhanced RECK cellular content (Figure 6).

### NF-κB nuclear content

**Figure 7:**
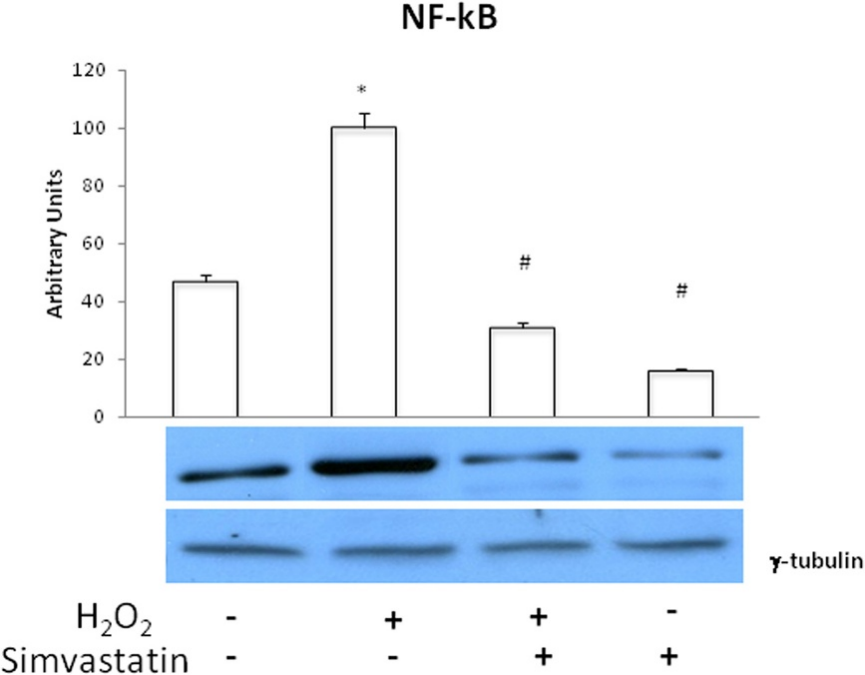
**NF-kB nuclear content.** Western blot evaluation of NF-κB nuclear levels in GLC-82 cells following or not H_2_O_2_ administration, in the presence or absence of 24 hours treatment with simvastatin. Data represent the mean ± s.e.m. of three experiments. *P < 0.01 (H_2_O_2_ vs control); #P < 0.01 (H_2_O_2_ + simvastatin and simvastatin vs H_2_O_2_).

H_2_O_2_ elicited a significant (P <0.01) increase in NF-κB nuclear levels, and this effect was significantly inhibited by treatment with simvastatin (P <0.01) (Figure 7).

### MyD88 and TRAF6 expression

**Figure 8:**
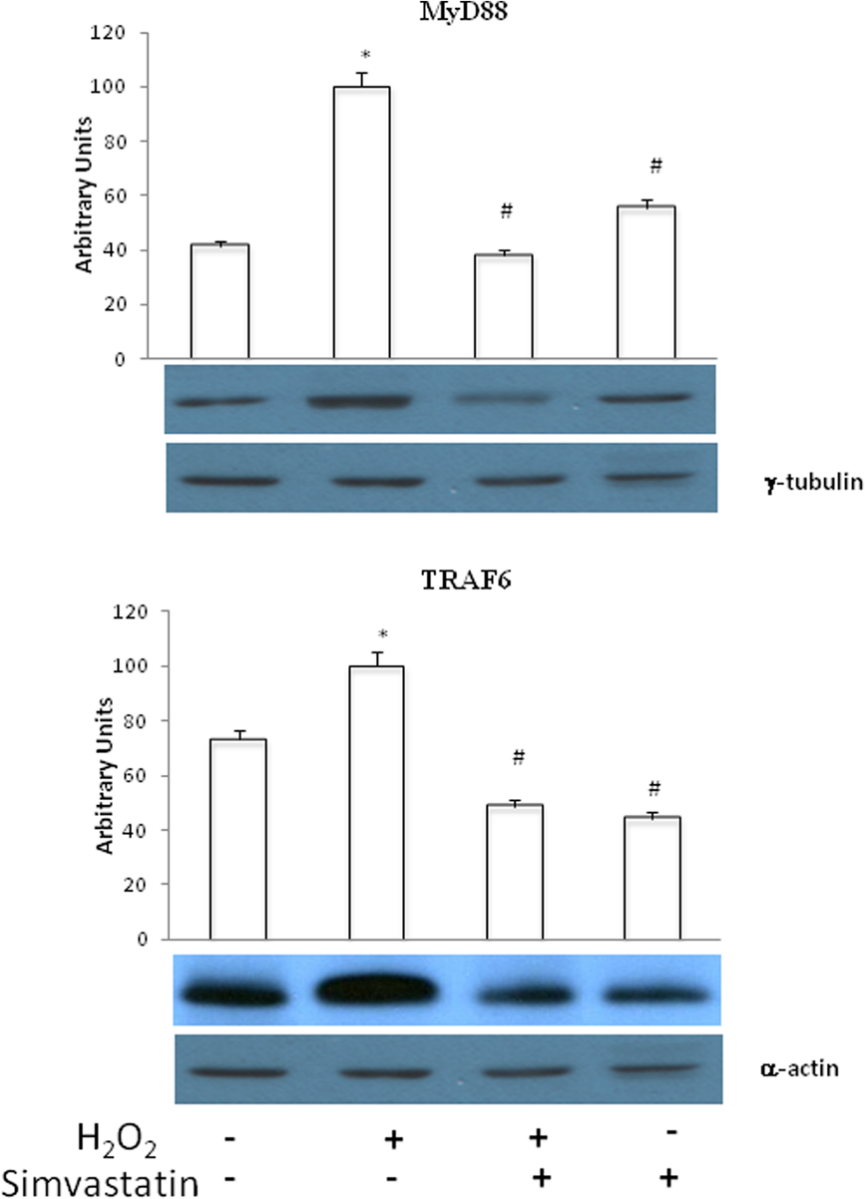
**MyD88 and TRAF6 expression.** Western blot evaluation of MyD88 (upper panel) and TRAF6 (lower panel) expression followingH_2_O_2_ administration, in the presence or absence of 24 hours treatment with simvastatin. Data represent the mean ± SEM of three experiments. *P <0.01 (H_2_O_2_ vs control); #P <0.01 (H_2_O_2_ + simvastatin vs H_2_O_2_).

H_2_O_2_ induced a significant (P < 0.01) increase in MyD88 and TRAF6 expression, and these effects were significantly (P <0.01) inhibited by treatment with simvastatin (Figure 8).

### TRADD and TRAF2 expression

**Figure 9:**
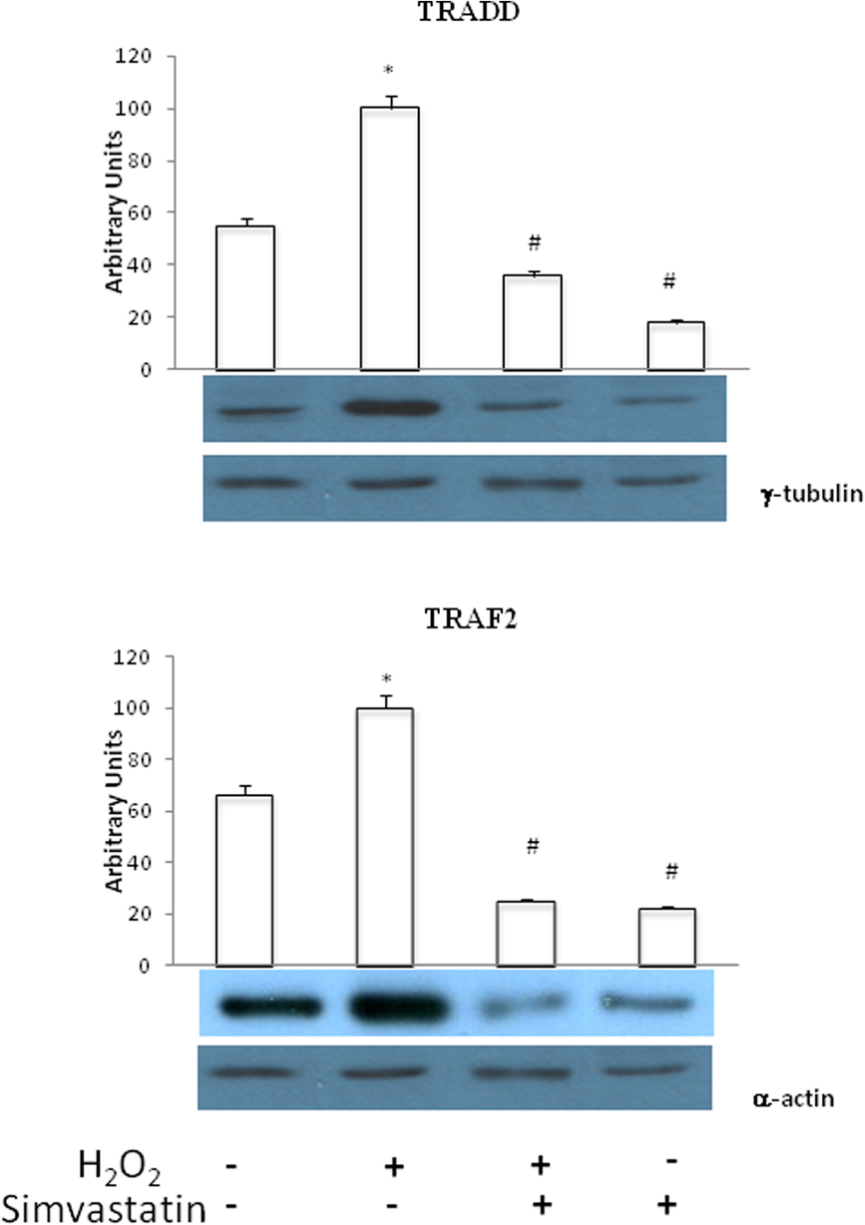
**TRADD and TRAF2 expression.** Western blot evaluation of TRADD (upper panel) and TRAF2 (lower panel) expression following or not H_2_O_2_ administration, in the presence or absence of 24 hours treatment with simvastatin . Data represent the mean ± SEM of three experiments. *P <0.01 (H2O2 vs control); #P <0.01 (H2O2 + simvastatin and simvastatin vs H2O2).

H_2_O_2_ caused a significant (P <0.01) increase in TRADD and TRAF2 expression. These effects were significantly (P <0.01) inhibited by treatment with simvastatin (Figure 9).

### IL-8 secretion

**Figure 10:**
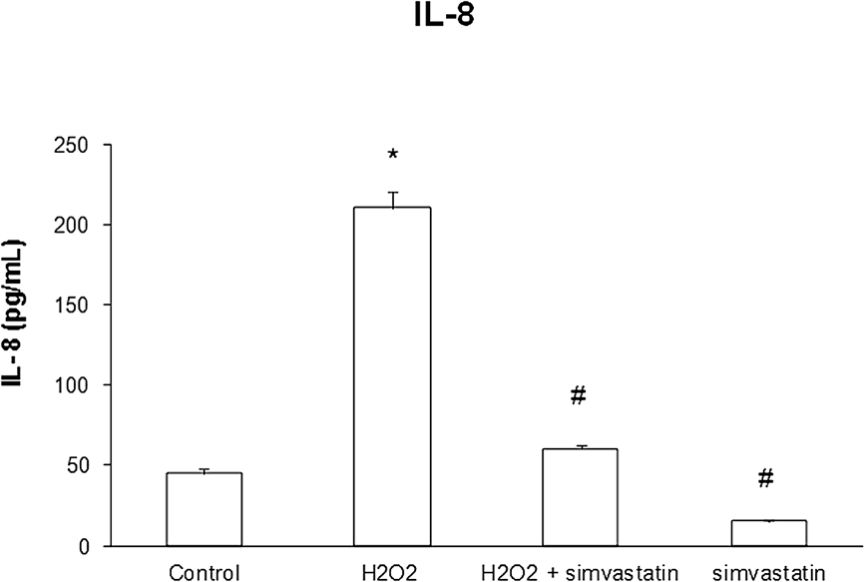
**IL-8 secretion.** ELISA evaluation of IL-8 secretion in GLC-82 cell culture supernatants in the presence or absence of exposure to H2O2 and with or withouta 24 hours treatment with simvastatin. Data represent the mean ± SEM of three experiments. *P <0.01 (H2O2 vs control); #P <0.01 (H2O2 + simvastatin and simvastatin vs H2O2).

H_2_O_2_ elicited a significant (P <0.01) increase in IL-8 secretion, which was significantly (P <0.01) prevented by pretreatment with simvastatin (Figure 10).

## Discussion

In this study we evaluated the effects of simvastatin on lung adenocarcinoma GLC-82 cells, exposed to hydrogen peroxide (H_2_O_2_). Interestingly, oxidants are capable of exerting a tumorigenic action via molecular mechanisms which at least in part are also activated by proinflammatory pathways [20, 21], and several authors have shown that both oxidative stress and inflammation are involved in the development and progression of cancer [22].

Therefore, we focused our attention on several cellular events triggered by oxidative stress. Within such a research context, it is noteworthy to point out that simvastatin was able to induce a comprehensive inhibition of many phenomena related to lung cancer.

In this regard, Manfredini et al. have previously reported that simvastatin is able to modulate oxidative DNA damage [23], and we also showed that simvastatin significantly decreased H_2_O_2_-induced cell proliferation rate, probably through inhibition of Ras-dependent phosphorylative activation of the ERK1/2 subgroup of MAPKs [13]. In fact, H_2_O_2_ dramatically enhanced ERK phosphorylation, and such a stimulatory action was markedly reduced by simvastatin. This implies that simvastatin affects ERK function by inhibiting mevalonate-dependent post-translational prenylation of Ras, thus impairing the sequential activation of the Ras/Raf/MEK/ERK signalling cascade [24]. Therefore, this mechanism may be also involved in the observed pro-apoptotic action of simvastatin, documented by the increased expression of active caspase-3 [25].

Interestingly, in our present study the anti-proliferative and pro-apoptotic effects of simvastatin were also paralleled by a drug-induced up-regulation of RECK protein, which plays a pivotal role in inhibiting matrix metalloproteinases. MMP-2 and MMP-9 are responsible for extracellular matrix degradation, and for the consequent neo-angiogenesis, vascular invasion and metastatic potential that characterize malignant tumours [26–28]. Indeed, in many cancers RECK down-regulation is associated with high levels of MMP-9 [9]. Otherwise, preservation of RECK expression in some neoplasms correlates with a relatively low microvascular density as well as with a better prognosis, due to a decreased tendency to metastatic invasion [29]. Consistently with these considerations, our results show that H_2_O_2_ down-regulated RECK in GLC-82 cells, whereas simvastatin restored its expression. Moreover, H_2_O_2_ enhanced MMP-2 and MMP-9 cellular expression, and these effects of oxidative stress were prevented by simvastatin.

Other important observations reported in the present study refer to further aspects of signal transduction. In particular, our data demonstrate that the noxious effects of oxidative stress on human lung adenocarcinoma cell lines, as well as the potential anti-cancer activity of statins, are also attributable to interferences with the signalling pathways linked to innate immunity [30]. In this regard, it is remarkable that H_2_O_2_ induced the expression of MyD88, a key molecular component of the intracellular signalling machinery activated by Toll-like receptors (TLRs), and once again simvastatin abrogated such effect. MyD88 associates with TLRs; upon stimulation, MyD88 recruits IL-1 receptor-associated kinase (IRAK) to TLRs via interaction of the death domains of both molecules [31]. IRAK is activated by phosphorylation and then associates with tumor necrosis factor (TNF) receptor-associated factor 6 (TRAF6). Similarly to MyD88, the opposite actions of H_2_O_2_ and simvastatin translated into significant increases and decreases in TRAF6 expression, respectively. Therefore, on the basis of this experimental work, we canspeculate that oxidative stress triggers a particular type of tissue injury which is sensed by the surveillance network of innate immunity via TLR stimulation. In fact, it has been demonstrated that H_2_O_2_ can prime the responsiveness of the innate immune system through a recruitment of TLRs to cell plasma membrane [32]. However, this chain of molecular events, including the essential step of MyD88 activation, ultimately culminates in the downstream involvement of the transcription factor NF-κB, which is a key effector of the carcinogenic and proinflammatory attitudes of oxidative stress. It can thus be argued that, by interrupting such complex molecular cascades, statins can have relevant anti-cancer and anti-inflammatory properties.

Other important components of the macromolecular complex associated with TNF receptor superfamily, whose stimulation leads to NF-κB activation, include TRADD and TRAF2 proteins [33]. Here it is shown that both TRADD and TRAF2 were susceptible to up-regulation induced by H_2_O_2_, and this effect was prevented by simvastatin. Because the above mentioned signaling pathways converge towards NF-κB activation, it was not surprising to detect in GCL-82 cells, as a result of their exposure to H_2_O_2_, an increased nuclear content of NF-κB. Indeed, nuclear translocation of this transcription factor is the essential step underlying its activation. Consistently with the overall experimental design of this study, it was well expected to find the observed decrease of NF-κB nuclear levels elicited by simvastatin.

Moreover, a further evidence of the opposite actions of H_2_O_2_ and simvastatin was found by analyzing, in GLC-82 cell culture supernatants, the changes referring to the concentrations of IL-8, which acts as a powerful chemoattractant for neutrophils [34]. In particular, IL-8 secretion was up-regulated and down-regulated by H_2_O_2_ and simvastatin, respectively. Indeed, this proinflammatory chemokine is encoded by a gene targeted by the transcriptional stimulatory activity of NF-κB [35].

Therefore, taken together these results strongly suggest the existence of a tight cross-talk involving the proliferative, metastatic and proinflammatory mechanisms activated by oxidative stress in GLC-82 cells. Moreover, our data clearly demonstrate that in this lung adenocarcinoma cell line simvastatin can effectively interfere with the complex signal transduction networks underlying such mechanisms. These findings confirm and further extend our previous data obtained in the same GLC-82 cell line, showing that simvastatin was able to significantly decrease cell proliferation and increase apoptosis via inhibition of the ERK1/2 subgroup of MAPKs [13].

With regard to MMP-2 and MMP-9, it is well known that these enzymes promote cancer progression through extracellular matrix and basement membrane degradation, resulting in the exposure of cryptic locations linked to invasion, metastasis and angiogenesis. The 5′ flanking regions of the MMP-2 and MMP-9 genes contain several functional regulatory motifs that bind transcription factors such as NF-κB [36, 37]. Through the interactions between NF-κB and its DNA binding sites, various agents including growth factors and cytokines are able to regulate MMP expression. Hence, the coordinated inhibitory effects of simvastatin on MMP2/9 expression and NF-κB activation imply the involvement of HMG-CoA reductase pathway in the process of metastatic invasion related to NF-κB and MMP activity. In fact, cancer cells usually express high levels of HMG-CoA reductase, which appear to be required by NSCLC cells to satisfy their increased need for isoprenoids and lipids.

Thus, by blocking Ras prenylation, simvastatin could interfere with NF-κB-dependent signaling networks responsible not only for MMP expression, but also for IL-8 synthesis. Indeed, it is reasonable to infer that, by inhibiting Ras/Raf-induced phosphorylative activation of MAPKs, simvastatin may be able to prevent IL-8 production elicited in GLC-82 cells by NF-κB activation due to oxidative stress.

## Conclusion

In conclusion, our results show that simvastatin is able to inhibit the effects of H_2_O_2_ on lung cancer cells, suggesting that simvastatin is able to induce an antiproliferative, pro-apoptotic and antinflammatory effects in presence of a pro-inflammatory mediator. These data could support the role of simvastatin in the prevention of cancer and lung inflammatory disease (i.e. chronic obstructive pulmonary diseases). In fact to date no definitive reports have been published regarding these diseases [38, 39]. However, in order to validate these preliminary observations further studies are needed, especially including controlled clinical trials.
